# Effect and Process Evaluation of a Cluster Randomized Control Trial on Water Intake and Beverage Consumption in Preschoolers from Six European Countries: The ToyBox-Study

**DOI:** 10.1371/journal.pone.0152928

**Published:** 2016-04-11

**Authors:** An-Sofie Pinket, Wendy Van Lippevelde, Ilse De Bourdeaudhuij, Benedicte Deforche, Greet Cardon, Odysseas Androutsos, Berthold Koletzko, Luis A. Moreno, Piotr Socha, Violeta Iotova, Yannis Manios, Marieke De Craemer, ToyBox-study group

**Affiliations:** 1 Department of Public Health, Ghent University, Ghent, Belgium; 2 Department of Movement and Sport Sciences, Ghent University, Ghent, Belgium; 3 Department of Human Biometry and Biomechanics, Vrije Universiteit Brussel, Brussels, Belgium; 4 Department of Nutrition and Dietetics, School of Health Science and Education, Harokopio University, Athens, Greece; 5 Ludwig-Maximilians-University of Munich, Dr. von Hauner Children’s Hospital, München, Germany; 6 GENUD (Growth, Exercise, NUtrition and Development) Research Group, University of Zaragoza, Zaragoza, Spain; 7 The Children’s Memorial Health Institute, Warsaw, Poland; 8 Dept. of pediatrics and medical Genetics, Medical University of Varna, Varna, Bulgaria; Karolinska Institutet, SWEDEN

## Abstract

**Background:**

Within the ToyBox-study, a kindergarten-based, family-involved intervention was developed to prevent overweight and obesity in European preschoolers, targeting four key behaviours related to early childhood obesity, including water consumption. The present study aimed to examine the effect of the ToyBox-intervention (cluster randomized controlled trial) on water intake and beverage consumption in European preschoolers and to investigate if the intervention effects differed by implementation score of kindergartens and parents/caregivers.

**Method:**

A sample of 4964 preschoolers (4.7±0.4 years; 51.5% boys) from six European countries (Belgium, Bulgaria, Germany, Greece, Poland, Spain) was included in the data analyses. A standardized protocol was used and parents/caregivers filled in socio-demographic data and a food-frequency questionnaire. To assess intervention effects, multilevel repeated measures analyses were conducted for the total sample and for the six country-specific samples. Based on the process evaluation questionnaire of teachers and parents/caregivers, an implementation score was constructed. To assess differences in water intake and beverage consumption by implementation score in the total sample, multilevel repeated measures analyses were performed.

**Results:**

Limited intervention effects on water intake from beverages and overall beverage consumption were found. However, important results were found on prepacked fruit juice consumption, with a larger decrease in the intervention group compared to the control group. However, also a decline in plain milk consumption was found. Implementation scores were rather low in both kindergartens and parents/caregivers. Nevertheless, more favorable effects on beverage choices were found in preschoolers whose parents/caregivers and kindergarten teachers had higher implementation scores compared to those with lower implementation scores.

**Conclusion:**

The ToyBox-intervention can provide the basis for the development of more tailor-made interventions. However, new strategies to improve implementation of interventions should be created.

## Background

European preschoolers do not meet the water standards [1–2]. However, water is a vital nutrient in life, since the optimal functioning of human body requires a good hydration level [3–4]. Even mild dehydration can lead to significant consequences, such as headache, lethargy, impaired concentration and decrease of cognitive performance [5–9]. Moreover, children are more vulnerable to dehydration [7]. Water can be extracted from plain water, but also from other beverages and food [10–11]. As only 20% of total water intake comes from food, water intake through beverages (i.e., plain water and other beverages) is the most important water source [11]. In addition, many preschoolers consume excessive amounts of sugared beverages [1,12–15]. A recent European study showed that nearly a quarter of total water intake from beverages was derived from sweetened beverages (such as fruit juices, soft drinks and sugared milk) and only slightly more than half of water intake from beverages was derived from plain water [1]. An excessive intake of added sugars, e.g., through soft drinks, can lead to energy imbalance and thus to overweight [16–17]. Since both quality and quantity of water intake may be a problem already at preschool age, interventions targeting water intake in preschoolers gain importance [1].

Parents play a fundamental role in developing a home environment that stimulates healthy eating habits among children through general parenting styles and parenting practices including availability and accessibility, role-modeling, monitoring, pressuring, restricting and rewarding regarding eating behaviours [18]. Parenting practices also influence the intake of soft drinks [19]. It is expected that also in preschoolers’ water intake and other beverage choices parents play an important role, but research is currently lacking. In addition, preschoolers also spend a lot of time at kindergartens. Healthy behaviours are promoted by stimulating and encouraging environments in which children live and play, such as kindergartens [20]. A kindergarten is the ideal place to promote water consumption and healthy beverage choices [20–21]. Interventions combining high levels of parental involvement and interactive school-based learning appeared to be highly effective in tackling obesity in preschoolers [21]. To our knowledge, interventions targeting water intake and beverage choices in preschoolers are scarce. Most interventions targeted older children and were aimed at reducing the intake of sugar-sweetened beverages rather than promoting water intake and healthy beverage choices.

One of the targeted key behaviours related to early childhood obesity in European preschoolers in the ToyBox-intervention was water consumption, next to snacking, physical activity and sedentary behaviour. The ToyBox-study (multifactorial evidence-based approach using behavioural models in understanding and promoting fun, healthy food, play and policy for the prevention of obesity in early childhood) was an EU-funded large-scale study in preschoolers (3.5–5.5 years old) and their families from six European countries (Belgium, Bulgaria, Germany, Greece, Poland, and Spain) [22]. Within the ToyBox-study, a kindergarten-based, family-involved intervention was developed, implemented and tested. The first purpose of this study was to examine the effects of the ToyBox-intervention on the water intake and beverage consumption in European preschoolers, including a large sample of preschoolers. A cross-European study may be more valuable than a study with a regional focus. The intervention effects were investigated in the total sample and in all six countries.

Research on process evaluation is a valuable addition to studying the effect evaluation. As interventions are not always implemented as intended, process evaluation can help to better understand the effects of a health promoting intervention [22]. Process evaluation can throw light on the mechanisms and processes responsible for the results and variation in results in the target group [23]. Knowing which aspects of the intervention were delivered and how well they were conducted is essential to make an accurate interpretation of outcomes. Next to kindergarten implementation results, obtaining insight into how parents perceived the intervention is important as involving parents in kindergarten-based interventions remains challenging [24]. Therefore, the second aim of this study was to investigate if the effects of the intervention differed by implementation score of the intervention at the kindergartens and at home.

## Methods

### Study protocol

The ToyBox-intervention was developed following the PRECEDE-PROCEDE model and the Intervention Mapping protocol [25]. The study had a randomized cluster design and consisted of a pre- and posttest design with intervention and control schools in all six European countries. The ToyBox-study (www.toybox-study.eu) is registered with the clinical trials registry clinical_trials.gov, ID: NCT02116296. The ToyBox-study was approved by Ethical Committees in all six European countries, in line with national regulations (i.e., the Ethical Committee of Ghent University Hospital (Belgium), Committee for the Ethics of the Scientific Studies (KENI) at the Medical University of Varna (Bulgaria), Ethikkommission der Ludwig-Maximilians-Universität München (Germany), the Ethics Committee of Harokopio University of Athens (Greece), Ethical Committee of Children’s Memorial Health Institute (Poland), and CEICA (Comité Ético de Investigación Clínica de Aragón (Spain)) [26]. Parents/caregivers were asked for written informed consent for the participation of their child in the study. A minimum sample of 800 children and their families and 20 kindergartens per country, resulting in a total sample of 4,800 children and their families and 120 kindergartens, was initially targeted. However, in order to account for an estimated dropout rate of about 30%, a minimum total number of about 6,500 children and their families were aimed to be recruited in the six participating countries. Detailed power calculations are described elsewhere [26]. The preschool children and their families were recruited at kindergartens, daycare centers or preschool settings, depending on the country regulations and legislation [26]. Precisely, in Germany, Bulgaria, Spain and Poland children/families were recruited from kindergartens, in Greece from kindergartens and daycare centers and in Belgium from preschool settings. In order to avoid confusion for the reader, all these settings (kindergartens, daycare centers, preschool settings) will be referred to as "kindergartens" in this paper. Kindergartens were recruited from different socio-demographic backgrounds within each of the provinces in the different countries. Lists of all municipalities that exist within the selected provinces were created with information on the socio-economic status (SES) variables of the municipalities. Tertiles including three different socio-demographic groups were created based on the selected SES variables, and each country randomly selected approximately five municipalities per SES status (low, medium and high SES). Then, kindergartens within these randomly chosen municipalities were randomly selected (with the exclusion of the lowest 20% of the kindergartens with the smallest number of pupils) [26]. The selected kindergartens were visited by researchers to inform the kindergarten staff about the ToyBox-study. After kindergartens agreed to participate in the study, preschoolers received an information letter to take home with information on the study for parents/caregivers. Subsequently, municipalities were randomly assigned to the intervention or the control condition (2:1) [26]. Kindergartens of the ToyBox-intervention group received the intervention, while kindergartens of the control group were asked to continue the normal kindergarten routine.

### The ToyBox-intervention: water-component

As mentioned previously, the ToyBox-intervention targeted four key behaviours related to early childhood obesity: water consumption, snacking behaviour, physical activity and sedentary behaviour. The timeplan of the ToyBox-intervention was designed so as to account for country-specific differences with regard to the opening and closing dates of the kindergartens and the duration and timing of national holidays. Recruitment of participants started in February 2012 and baseline data was collected between May and June 2012. The entire ToyBox-intervention lasted from September 2012 until March 2013 for 24 weeks in the school year 2012–2013, with the drinking behaviour module implemented in weeks 1 to 4 and a repetition period in weeks 17 and 18. However, some environmental changes, such as water stations, were implemented during the whole school year. Follow-up evaluation was performed between May and June 2013 [26].

Prior to the intervention, teachers were invited to two teacher training sessions in which researchers explained the ToyBox-intervention and the materials. A third teacher training session was planned before the repetition period [27–28]. The participation of the teachers in the training sessions was not compulsory. Nevertheless, the participation of all intervention teachers was aimed for. However, to ensure representation of all intervention classes, it was aimed that at least one teacher per class attended each training session. In case none of the teachers of one class was able to attend a session, they were invited again on one of the following days when the session they missed was repeated. Teachers were not tested, but researchers provided certificates of attendance to the teachers as incentives. No teachers were excluded from the intervention because of absence during the training sessions, they could still deliver the intervention based on the provided ToyBox-manuals.

A box including the ToyBox-intervention material was provided to teachers of the intervention group to implement the intervention. Since this study focuses on water consumption, the description of the intervention materials will also focus on this component. Information on the other components of the intervention are described elsewhere [26]. The box contained a teacher’s guide with general information on the ToyBox-intervention and the importance of water consumption, a classroom activity guide on water consumption and a kangaroo hand puppet. The classroom activity guide regarding water consumption consisted of three sections: setting environmental changes in the classroom (installing a water drinking station), preschoolers implementing the actual behaviour (e.g. drinking water regularly) and teachers implementing fun classroom activities (3 kangaroo stories, 3 sensory perception games, 2 experiments and 3 excursions) aiming at total class participation. The environmental changes were conducted before the start of the intervention and retained throughout the whole school year, while the implementation of the actual behaviour was also performed throughout the whole school year.

Parents/caregivers were also involved in the intervention. Therefore, preschoolers received two newsletters and two tip-cards and a poster to take home, all containing information and tips regarding preschoolers’ water consumption. The first newsletter and tip-card focused on drinking enough water, while the second newsletter and tip-card focused on making healthy beverage choices.

### Measures

#### Water intake and beverage consumption

Both in the baseline and follow-up measurements, parents/caregivers were asked to describe the child’s usual food and beverage habits over the last 12 months in a food frequency questionnaire (FFQ) for young children, which was developed based on a previously validated FFQ by Huybrechts et al. [29]. Results of the validation of the FFQ by Huybrechts and colleagues showed moderate to good reproducibility (ICC ranged from 0.62 to 0.79) and good relative validity (Spearman correlation ranged from 0.56 to 0.65) for beverage intake [29]. Only the items about beverage consumption were used in this study. These items were plain water, soft drinks, light soft drinks, home-made and freshly squeezed fruit juice, pre-packed/bottled fruit juice, tea, smoothies (all kinds), plain milk and sugared or chocolate milk. For each of these beverages, the frequency of consumption was asked. Response categories were: “never or less than once per month”, “1–3 days per month”, “1 day per week”, “2–4 days per week”, “5–6 days per week” and “every day”. Next, the average consumption per day was asked. The response categories were “100ml or less”, “100–200ml”, “200–300ml”, “300–400ml”, “400–500ml”, “500–600ml”, “600–700ml”, “700–800ml”, “800–900ml”, “900–1000ml” and “1000ml or more”. From these data, the average amount of the different beverages in ml per day was calculated by multiplication of number of days per week and amount per day in ml (using the midpoint) divided by 7 (total number of days in a full week). The water intake from these beverages was calculated based on the Dutch food composition database [30], by multiplication of the average intake in ml a day and the amount of water per ml of each beverage.

#### Socio-demographic variables

Sex and date of birth were reported by one of the parents/caregivers of the preschoolers in the primary caregivers questionnaire. Preschool children’s age was computed based on the date of birth and the date when the questionnaire was completed. Education of the parents/caregivers was self-reported in the primary caregivers questionnaire. The education level of the mother was used as SES indicator. Education level has been identified as an important indicator for SES [31]. Education level was dichotomized into lower (14 or fewer years of education) and high (more than 14 years of education) SES, which distinguishes families with a mother who has completed medium or higher education, college or university training from other families [32].

All questionnaires are available on the ToyBox-website (www.toybox-study.eu) and in the second ToyBox supplement issue [33].

### Process evaluation

**Fig 1:**
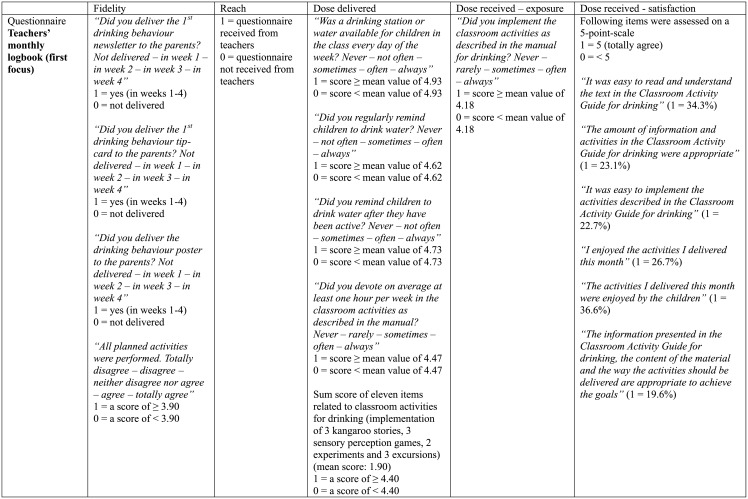
Overview process evaluation questions to calculate the implementation score (total score on 30) for teachers (first focus).

**Fig 2:**
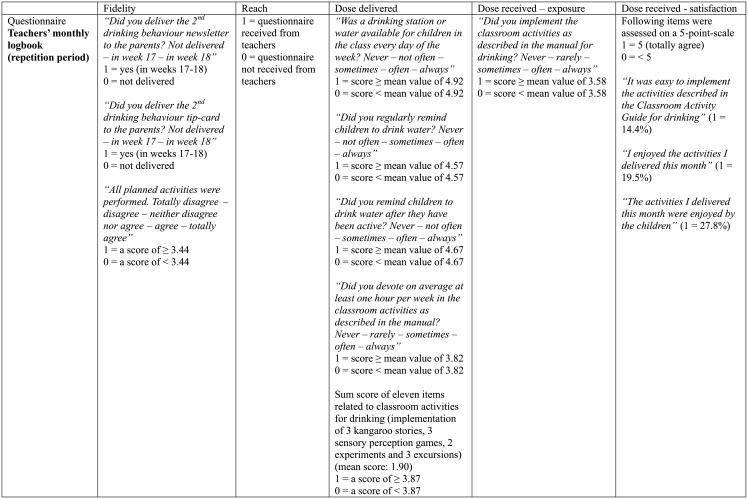
Overview process evaluation questions to calculate the implementation score (total score on 30) for teachers (repetition period).

**Fig 3:**
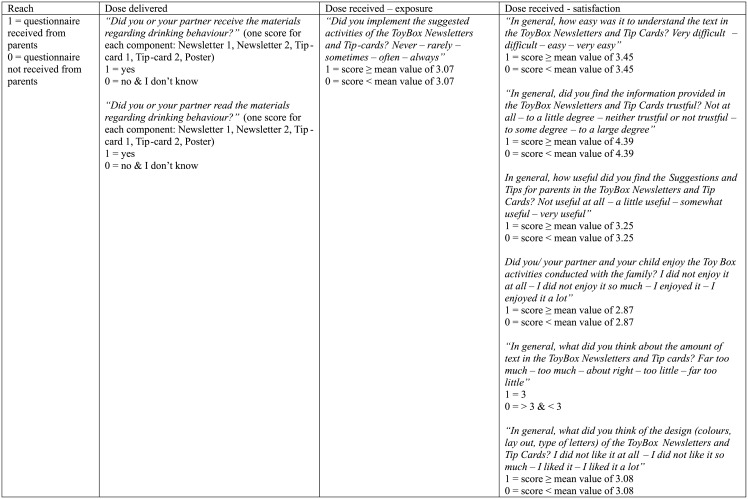
Overview process evaluation questions to calculate the implementation score (score on 18) for parents/caregivers.

Both teachers and parents/caregivers of the intervention group were asked to complete a process evaluation questionnaire, to get insight in the role of these two main implementers. Teachers received six monthly logbooks and parents/caregivers were asked to complete an implementation questionnaire at the end of the intervention [34]. The process evaluation has been performed according to the model of Saunders et al. which targets process evaluation of health promoting interventions [22]. The model contains several key elements: fidelity, reach, dose delivered, dose received—exposure, and dose received—satisfaction [22]. These key elements can be found in Figs 1 and 2 (for teachers) and Fig 3 (for parents/caregivers). For each element, questions of the process evaluation questionnaire are displayed with the information on the coding process. The internal consistency of the key elements of the process evaluation score for parents/caregivers was moderate to good (Cronbach’s α dose delivered = 0.94 and Cronbach’s α satisfaction = 0.61), while the internal consistency of the key elements of the process evaluation score of teachers ranged from borderline acceptable to good (Cronbach’s α fidelity = 0.42; Cronbach’s α reach = 0.51; Cronbach’s α dose delivered = 0.66; Cronbach’s α exposure = 0.40 and Cronbach’s α satisfaction = 0.86). All specific questions were scored 0 or 1 depending on the answers of the parents/teachers [24]. A total implementation score was computed for both teachers and parents/caregivers, with a minimum score of 0 and a maximum score of 30 for teachers and 18 for parents/caregivers. A minimum score of 0 indicated that the process evaluation questionnaire was not filled in, as ‘reach’ was scored 1 if the questionnaire was received from the teachers or parents/caregivers, leading to a minimum score of 1 if the questionnaire was completed. Preschoolers with an implementation score of 0 were deleted from the process evaluation analysis. The total implementation score represents the level of implementation of the intervention in the kindergarten classes and at home. For each kindergarten, a mean score was computed from the different teacher scores as to achieve one score for each kindergarten.

A higher score represents a higher level of intervention implementation. Based on the median of the implementation score of the parents/caregivers and kindergartens, preschoolers were divided into two groups: a low and high level of implementation. Next, a combined implementation score was constructed, creating four groups: preschoolers with a low kindergarten/low parental implementation score (n = 1514), high kindergarten/low parental implementation score (n = 1584), low kindergarten/high parental implementation score (n = 1343) and high kindergarten/high parental implementation score (n = 1080). A significant correlation between parent/caregiver and school implementation scores was found, however the association was very small (r = -0.06, p<0.001).

Only preschoolers with both parental and kindergarten implementation scores were included in the analyses. Preschoolers of the control group were added as a fifth group (n = 3428) to investigate the effect of implementation level on water intake and beverage consumption.

### Statistical analyses

Descriptive statistics were performed using IBM SPSS Statistics for Windows, version 21.0 (Amonk, NY: IBM Corp). Analysis of variance (ANOVA) was used to assess country differences in implementation scores.

#### First aim

Multilevel repeated measures analyses were performed using MLWiN, version 2.30 (Centre for Multilevel Modelling, University of Bristol, UK) to assess the effectiveness of the intervention on water intake and beverage consumption. To take clustering of baseline and follow-up measurements of preschool children in kindergarten classes in kindergartens into account, multilevel modeling was used. Four levels were used: time, preschooler, kindergarten class and kindergarten. A three-way interaction between time, group and country was performed and significant differences by country were found. So, intervention effects were tested in the total sample and by country. To perform analysis in the total sample a fifth level was added (i.e., country). The analyses were adjusted for age, sex and SES. To correct for multiple testing the significance level was set at p<0.01

#### Second aim

To assess the differences in water intake and beverage consumption by implementation score, multilevel repeated measures analyses were performed. The differences were tested in the total sample and five levels (time, preschooler, kindergarten class, kindergarten and country) were used to take clustering into account. The analysis was adjusted for age, sex and SES. To correct for multiple testing the significance level was set at p<0.01

## Results

### Population characteristics

The total and country-specific population characteristics are presented in Table 1. The total sample included 4964 preschoolers (mean age 4.7 ± 0.4 years, 51.5% boys), 38.5% had a mother with a lower level of education (≤ 14 years of education). Significant differences in SES were found between the different countries (F = 53.31; p<0.001). The Polish sample counted the lowest percentage of preschoolers of lower SES mothers (20.7%), the Greek sample the highest (51.4%).

**Table 1:** Characteristics of participants of the total sample and each country separately.

	Total	Belgium	Bulgaria	Germany	Greece	Poland	Spain
N	4964	767	643	882	824	1021	827
Age	4.7±0.4	4.4±0.5	4.9±0.3	4.5±0.5	4.9±0.3	4.9±0.3	4.9±0.3
Gender (% male)	51.5	50.8	49.8	51.8	50.5	50.9	54.5
SES (years of school education mother), % lower SES (= % ≤14 years of education)	38.5	34.1	40.5	51.4	52.9	20.7	34.8
Mean implementation score kindergartens	16.3±5.4	9.9±3.1	19.6±5.1	14.0±4.4	17.8±4.5	20.2±4.4	16.4±3.9
Mean implementation score parents/caregivers	9.5±5.3	9.1±5.4	11.5±4.6	10.5±4.3	11.2±5.0	5.9±5.1	10.7±4.5

### Intervention effects on water intake and beverage consumption

Results obtained from the multilevel repeated measures analyses for the total water intake and beverage consumption in the total sample are shown in Table 2. Only for prepacked fruit juice (p<0.001) significant intervention effects were found in the total sample. In both the intervention and control group the prepacked fruit juice consumption decreased, with a larger decrease in the intervention group (mean difference: -33ml) compared to the control group (mean difference: -10ml).

**Table 2:** Intervention effects for total water and all beverages in the total sample (adjusted for age, sex, SES and country).

	Group	Mean Baseline	Mean Follow-up	Time*Condition β
Plain water	I	549	583	11.0
	C	542	564	
Tea	I	49	48	-3.6
	C	50	52	
Soft drinks	I	67	54	-4.9
	C	66	57	
Light soft drinks	I	9	11	1.0
	C	9	10	
Pure fruit juice^†^	I	49	46	-1.7
	C	53	52	
Prepacked fruit juice^‡^	I	112	79	-23.5**
	C	106	96	
Smoothies	I	17	16	2.0
	C	19	16	
Plain milk	I	219	201	-12.0
	C	219	213	
Sugared and chocolate milk	I	44	36	0.9
	C	49	40	
Total Water^§^	I	1059	1024	-26.7
	C	1053	1046	

^†^ Home-made, freshly squeezed

^‡^ Pre-packed/bottled

^§^ Calculated from the water content from the various beverages.

*P<0.01

**P<0.001

I = intervention group; C = control group

Results obtained from the multilevel repeated measures analyses for the total water intake and beverage consumption in the six country-specific samples are shown in Tables 3 and 4. In Belgian preschoolers, significant intervention effects for plain milk consumption were found (p<0.001). In both the intervention and control group a decrease of plain milk consumption was found. However, a larger decrease was seen in the intervention group (mean difference: -49ml) than in the control group (mean difference: -1ml). No other significant intervention effects were found in Belgian preschoolers.

**Table 3:** Intervention effects for total water and all beverages in Belgium, Bulgaria and Germany (adjusted for age, sex and SES).

		Belgium			Bulgaria			Germany		
	Group	Mean Baseline	Mean Follow-up	Time*Condition β	Mean Baseline	Mean Follow-up	Time*Condition β	Mean Baseline	Mean Follow-up	Time*Condition β
Plain water	I	357	412	49.0	666	587	-86.5	462	524	31.8
	C	375	381		640	648		443	473	
Tea	I	6	6	0.1	64	63	1.1	62	58	-12.7
	C	8	8		79	78		57	66	
Soft drinks	I	89	84	12.2	33	24	-4.8	46	42	-4.1
	C	109	91		29	24		51	52	
Light soft drinks	I	23	26	-0.2	12	12	-1.3	5	6	0.4
	C	23	26		12	12		5	5	
Pure fruit juice^†^	I	18	15	-4.1	44	42	-1.8	57	43	6.0
	C	17	18		46	46		70	51	
Prepacked fruit juice^‡^	I	92	67	-8.8	90	49	-18.2	121	76	-46.9**
	C	103	87		77	55		110	112	
Smoothies	I	2	2	0.1	11	9	-4.0	2	3	-0.4
	C	3	3		7	9		2	3	
Plain milk	I	190	141	-47.4**	100	92	9.2	98	89	-5.3
	C	172	171		92	76		99	95	
Sugared milk	I	91	73	-0.2	34	28	-2.6	26	23	-3.0
	C	103	85		26	23		29	29	
Total Water^§^	I	814	784	6.0	1017	878	-106.4	839	832	-28.2
	C	852	815		975	943		824	844	

^†^ Home-made, freshly squeezed

^‡^ Pre-packed/bottled

^§^ Total water from beverages: calculated from the water content from the various beverages.

*P<0.01

**P<0.001

I = intervention group; C = control group

**Table 4:** Intervention effects for total water and all beverages in Greece, Poland and Spain (adjusted for age, sex and SES).

		Greece			Poland			Spain		
	Group	Mean Baseline	Mean Follow-up	Time*Condition β	Mean Baseline	Mean Follow-up	Time*Condition β	Mean Baseline	Mean Follow-up	Time*Condition Β
Plain water	I	638	664	-16.9	423	518	49.6	771	788	21.9
	C	657	699		382	427		790	785	
Tea	I	7	5	-4.1	156	156	-3.5	4	5	1.0
	C	6	8		155	158		2	2	
Soft drinks	I	13	10	-3.7	212	163	-26.6	19	19	0.2
	C	9	10		191	168		18	18	
Light soft drinks	I	4.	4	-0.2	8	14	7.1	5	4	-0.9
	C	3	3		10	10		5	5	
Pure fruit juice^†^	I	111	106	-7.8	29	33	5.8	37	37	-13.0*
	C	120	122		30	29		36	49	
Prepacked fruit juice^‡^	I	73	49	-15.0	206	165	-25.0	91	67	-20.7
	C	76	66		194	178		79	77	
Smoothies	I	8	9	1.9	36	34	1.8	47	41	10.2
	C	8	7		41	37		56	40	
Plain milk	I	402	362	-39.0	186	187	6.1	340	334	4.2
	C	426	424		170	165		368	358	
Sugared milk	I	12	9	1.8	32	28	1.2	76	61	3.2
	C	10	5		36	30		92	73	
Total Water^§^	I	1200	1155	-76.4	1210	1129	20.1	1319	1291	7.6
	C	1243	1274		1133	1132		1367	1332	

^†^ Home-made, freshly squeezed

^‡^ Pre-packed/bottled

^§^ Total water intake from beverages: calculated from the water content from the various beverages.

*P<0.01

I = intervention group; C = control group

In German preschoolers, only for prepacked fruit juice consumption a significant intervention effect was found (p<0.001). In the intervention group, a decrease of prepacked fruit juice intake was found from baseline tot follow-up (mean difference: -45ml). In the control group, an increase of prepacked fruit juice consumption was seen from baseline to follow-up measurements (mean difference: +2ml).

In the Spanish sample, a significant intervention effect was found for pure fruit juice (p = 0.008). An increase of pure fruit juice intake was seen in preschoolers of the control group (mean difference: +13ml), while no difference was found in the intervention group. No other significant intervention effects were found for Spanish preschool children.

In the Bulgarian, Greek and Polish sample, no significant intervention effects were found.

### Process evaluation results

The mean implementation score for kindergartens was 16.3±5.4 on a total of 30. A significant difference was found by country (F = 768.40, p<0.001). The mean implementation scores of the country-specific samples are presented in Table 1. A low implementation score for kindergartens, based on the median, ranged from 1.0 to 17.0, a high score ranged from 17.1 to 30.0.

The mean implementation score for parents/caregivers was 9.5±5.3 on a total of 18. A significant difference was found by country (F = 195.07, p<0.001). The mean implementation scores of the country-specific samples are presented in Table 1. A low parental implementation score, based on the median, ranged from 1.0 to 11.0, a high score ranged from 11.1 to 18.0.

**Fig 4:**
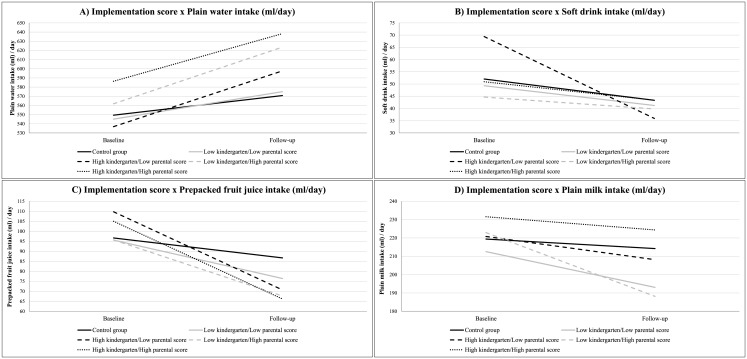
Process evaluation results: Implementation score x Plain water intake, Soft drink intake, Prepacked fruit juice intake and Plain milk intake (ml/day).

Fig 4 shows the most important results on the differences in water intake and beverage consumption from baseline to follow-up by implementation score in the total sample. The findings are discussed below.

Regarding plain water (Fig 4A), a significant interaction effect was found. In both preschoolers with a high kindergarten/low parental and low kindergarten/high parental implementation score and preschoolers of the control group a significant increase in plain water consumption was found. A larger increase was found in preschoolers with a high kindergarten/low parental (β = 60.51, p<0.001, mean difference: +61ml) and low kindergarten/high parental (β = 61.04, p<0.001, mean difference: +58ml) implementation score compared to preschoolers from the control group (β = 22.22, p = 0.006, mean difference: +22ml).

For soft drinks (Fig 4B), significant interaction effects were found. Preschoolers with a high kindergarten/low parental implementation score significantly decreased their soft drink consumption (β = -33.46, p<0.001, mean difference: -34ml), while no significant change was found in preschoolers with a low kindergarten/low parental implementation score, low kindergarten/high parental implementation score, and high kindergarten/high parental implementation score. Also, in both preschoolers from the control group and preschoolers with a high kindergarten/low parental implementation score a significant decrease in soft drinks consumption was found, with a larger decrease in preschoolers with a high kindergarten/low parental implementation score (β = -33.46, p<0.001, mean difference: -34ml) compared to preschoolers from the control group (β = -8.45, p = 0.006, mean difference: -9ml).

Regarding prepacked fruit juice (Fig 4C), significant interaction effects were found. In preschoolers with a high implementation score for kindergarten, parents or both and preschoolers from the control group, a significant decrease in prepacked fruit juice consumption was found, with a higher decrease in preschoolers with a high implementation score for kindergartens (β = -38.91, p<0.001, mean difference: -39ml), parents (β = -28.28, p<0.001, mean difference: -28ml) or both (β = -38.26, p<0.001, mean difference: -38ml) compared to the control group (β = -9.93, p = 0.006, mean difference: -10ml). Also, in preschool children with a high kindergarten/low parental and a high kindergarten/high parental implementation score and preschoolers with low kindergarten/low parental implementation scores, a decrease of prepacked fruit juice intake was seen, with a higher decrease in preschoolers with a high kindergarten/low parental (β = -38.91, p<0.001, mean difference: -39ml) or high kindergarten/high parental implementation score (β = -38.26, p<0.001, mean difference: -38ml) compared to preschoolers with both low kindergarten and parental implementation scores (β = -19.53, p<0.001, mean difference: -20ml).

For plain milk (Fig 4D), significant interaction effects were found. Preschoolers with a low kindergarten/high parental implementation score had a significant decrease in plain milk consumption (β = -34.34, p<0.001, mean difference: -34ml), while no significant change in plain milk consumption was found in preschoolers with a high kindergarten/low parental implementation score. Also, preschoolers with a low kindergarten/high parental implementation score had a significant decrease in plain milk consumption (β = -34.34, p<0.001, mean difference: -34ml), while no significant change in plain milk consumption was found in preschoolers with high kindergarten/high parental implementation scores.

## Discussion

The aim of this study was to examine the effect of the ToyBox-intervention on water intake and beverage consumption in European preschoolers and to investigate if the effect of the intervention differed by implementation score of parents/caregivers and kindergartens. The Toybox-intervention was implemented in kindergartens, but also parents/caregivers were involved. It was expected that the intervention would increase the intake of water from beverages and plain water and decrease the consumption of sugared beverages (such as soft drinks, fruit juices and sugared milk). However, no intervention effects were found on plain water consumption and total water intake from beverages. For plain milk consumption, negative intervention effects were found in Belgian preschoolers, with a steeper decrease in plain milk consumption in preschoolers of the intervention group compared to the control group. A possible explanation for this might be that the ToyBox-intervention was not implemented as intended in Belgium, given the rather low results on parental implementation scores and the very low scores on kindergarten implementation score in the Belgian sample. Schools (including preschools) in Flanders already for many years implement health promotion strategies, so teachers might be less motivated to implement addition programs. Also similar findings were seen regarding differences in beverage consumption from baseline to follow-up by implementation score of parents/caregivers and kindergartens. Plain water consumption increased from baseline to follow-up in preschool children with high parental or high kindergarten implementation scores, while plain milk consumption decreased in preschoolers with a high parental implementation score. Parents/caregivers might have misunderstood the message on beverage consumption. However, preschoolers should consume 500ml milk a day to reach their recommended calcium intake. This norm also includes yoghurt and derivatives [35]. So milk should be a major source of water intake in this age group, in addition to plain water [36]. As was done in the ToyBox-study, messages to parents should make clear that it is not only important to make healthy beverage choices, but also that the quantity of total water intake from beverages should be kept in mind. Moreover, during the implementation of an intervention on water intake and beverage choices, special attention should be given to increase the total intake of healthy beverages instead of replacing one healthy beverage by another.

Also limited intervention effects were found on the consumption of sugared beverages. The intervention did not induce a significant decrease in soft drinks and sugared milk consumption. However, some positive intervention effects were found. Pre-packed/bottled fruit juice intake decreased more in the intervention group compared to the control group. These results were found in the total and in the German sample. In addition, the most important results on differences in beverage consumption from baseline to follow-up by the combined implementation level in kindergartens and parents/caregivers were seen for soft drinks and especially prepacked fruit juices. Regarding soft drinks, especially high kindergarten implementation scores seemed to be important to obtain a reduction in soft drink consumption. Regarding prepacked fruit juice, the consumption decreased in preschool children with a high parental implementation score, a high kindergarten implementation score and high scores on both compared to preschoolers with low scores or from the control group. This is an important finding, since a study on water intake and beverage consumption in European preschoolers using the baseline data of the ToyBox-study found that soft drink consumption of preschoolers appeared to be more limited than expected, while fruit juices appeared to be a larger problem than expected [1]. Fruit juices also contain a lot of sugar. Although these are mainly fruit sugars instead of artificially added sugars, fruit juices are not recommended as healthy water sources given the extra calories they supply [37]. It might be that more parents are already aware of the adverse health effects of soft drinks but still have insufficient knowledge about the high sugar content of fruit juices [38]. This finding was also reported in a study with focus group discussions with preschoolers’ parents in different European countries, where parents seemed to have a misconception about whether or not fruit juices are a healthy choice [39]. Therefore, a decrease in prepacked fruit juice consumption due to the ToyBox-intervention is definitely a positive finding. A study of Rader and colleagues (2014) also indicated that future interventions should not only focus on sugar-sweetened beverages to reduce calorie intake form beverage consumption, but also on fruit juices, since these are important contributors of caloric intake from beverages, especially in young children [38].

Overall, the process evaluation showed poor implementation scores. For instance, not all planned activities were performed in the kindergartens and a large part of the parents/caregivers did not read the newsletters and tip-cards or they did not implement the suggested activities of the newsletters and tip-cards. This could be a possible explanation for the limited intervention effects that were found. A previous review, based on results from over 500 studies, indicated that expecting perfect implementation is unrealistic [40]. An implementation level of 60% was already considered a positive result and only few studies attained implementation levels greater than 80% [40]. In the present study, the overall implementation in both kindergartens and households was lower than 60% (mean implementation score divided through maximum score). In three out of six countries kindergartens obtained 60% of the intended implementation, while in four out of six countries this level was obtained at the home environment. So, the level of implementation was not that low compared to earlier intervention studies. Nevertheless, future interventions should definitely take this into account and create efficient or innovative implementation strategies to improve intervention effects. As highlighted before, parents play a fundamental role in developing a healthy home environment [18]. As in a lot of previous interventions, in the present study parents/caregivers were only passively involved in the intervention through newsletters, tip-cards and posters via their child’s kindergarten [41–42]. Unfortunately, while the school or kindergarten area is a promising public health intervention setting, it has been found that parents are difficult to reach that way [43]. The implementation results of this study for instance show that a large part of the parents/caregivers do not read the newsletters and tip-cards. This could also be a possible explanation for the limited intervention effects. However, despite the poor implementation scores, the current study showed better effects in preschoolers whose parents/caregivers had higher implementation scores compared low implementation scores. Actively involving parents might be a promising strategy and might lead to even better intervention effects [44]. Studies show that modeling is an effective method to teach parents parenting skills, e.g. by providing videos with specific case studies about parenting skills towards drinking behaviour of young children [45]. In addition, the ToyBox-intervention targeted four behaviours instead of only focusing on drinking behaviour of preschoolers. A recent study of Kunin-Batson and colleagues (2015) indicated that parents prefer to focus on only one healthy child behaviour instead of several health behaviours [46]. Also, the intensity (a minimum of one hour a week) might have been too limited and the time spent on drinking behaviour (a total of six weeks) might have been too short. However, as highlighted before, the results on the process evaluation indicated that better implementation levels of the ToyBox-study in kindergartens and at home led to better results in plain water consumption, prepacked fruit juice consumption and soft drink intake. Therefore, the ToyBox-intervention can be used to target beverage consumption in preschoolers and high levels of implementation should be encouraged to create the best possible outcome. A possible explanation for country-differences in implementation levels could be found in the amount of existing health promotion activities prior to the ToyBox-intervention [47]. The lowest implementation levels at kindergartens were found in countries with the most existing health promotion activities (Belgium, Germany and Spain) and the highest implementations scores were found in countries where no prior activities were conducted (Poland and Bulgaria). So, possibly, kindergartens in the countries with a higher health promotion burden were less motivated to collaborate in another new intervention. No clear explanations could be found for the differences in implementation results between kindergartens and parents. Future studies should also focus on finding the underlying mechanisms of implementation in different environments. As indicated in the review of Durlak and DuPre (2008), many factors can affect implementation, such as organizational functioning and the level of technical assistance [40]. Future research on the influencing factors could yield interesting insights in understanding these underlying mechanisms of intervention implementation and would be useful for the planning of future intervention developers.

The current study holds some limitations that need to be acknowledged. We acknowledge that the ToyBox-sample is not a fully representative European sample, due to sampling in specific regions in each country. Samples included preschoolers of both low, medium and high SES backgrounds. The samples can give a fair approximation of the average situation in each country. The procedure of sampling in specific regions has also been used in several other European studies such as HELENA and ENERGY [48–49]. The process evaluation was measured by self-report. Participants may not be aware of intervention delivery or reception. More objective indicators of program delivery (e.g., observations), could be used in future interventions to avoid this limitation [50]. Because of the large number of statistical tests used in this study, bias may have occurred. However, to correct for multiple testing the significance level was set at p<0.01. Strengths of the present study include the large sample of preschoolers from six European preschoolers and the cluster randomized pre- and posttest design including an intervention and control group.

## Conclusion

The ToyBox-intervention had limited intervention effects on water intake and beverage consumption in preschoolers. However, the ToyBox-intervention induced a larger decrease in prepacked fruit juice consumption in the intervention group compared to the control group. Since excessive prepacked fruit juice consumption is a major problem in preschoolers, this is an important finding. However, a decline in plain milk consumption was also found. Implementation scores were both in kindergartens and parents/caregivers rather low. Nevertheless, the current study showed better effects in preschoolers whose parents/caregivers and kindergarten teachers had high implementation scores compared to preschoolers of low-implementers. The ToyBox-intervention can provide the basis for the development of more tailor-made interventions. New strategies to improve implementation of interventions should be created, such as participatory intervention development. In addition, the results of the process evaluation can also be used during future interventions to improve implementation.

## Supporting Information

S1 File Data file manuscript. (XLSX)
